# Impact of alpha-ketoglutarate treatment on enhancing vase life of chrysanthemum by enhancing antioxidant systems and suppressing ethylene biosynthesis

**DOI:** 10.1038/s41598-026-55945-4

**Published:** 2026-05-30

**Authors:** Parviz Malekzadeh, Soheila Samadi, Elham Ghasemifar

**Affiliations:** 1 Department of Biology, Faculty of Sciences, University of Qom, Qom, Iran; 2 Department of Biology, Payame Noor University (PNU), PO BOX 19395-3697, Tehran, Iran

**Keywords:** Antioxidant system, Alpha-ketoglutarate, Chrysanthemum, Ethylene biosynthesis, Vase life, Water balance, Biochemistry, Plant sciences

## Abstract

Chrysanthemum is a globally valued cut flower with substantial commercial importance; however, its postharvest longevity is often limited by rapid senescence. This study investigated the application of alpha-ketoglutarate (AKG), a cost-effective and environmentally friendly metabolic intermediate, for extending the vase life of cut chrysanthemum flowers. The effects of 5 mM AKG (compared to a 0 mM control) on vase performance, physiological traits, biochemical composition, and the molecular regulation of ethylene biosynthesis were systematically evaluated. Our results demonstrated that 5 mM AKG significantly prolonged vase life by improving water balance and enhancing flower hydration through increased water uptake. The treatment effectively reinforced the antioxidant defense system by increasing the contents of glutathione, ascorbic acid, total phenolics, and flavonoids, while stimulating the activities of catalase (CAT) and superoxide dismutase (SOD). Consequently, AKG inhibited the accumulation of reactive oxygen species (ROS), leading to a marked reduction in malondialdehyde (MDA) levels, electrolyte leakage (EL), and lipoxygenase (LOX) activity. Furthermore, AKG application suppressed ethylene (ETH) production by downregulating the activities of 1-aminocyclopropane-1-carboxylic acid synthase (ACS) and oxidase (ACO), which was further confirmed by the reduced expression of *CmACS1* and *CmACO1* genes. Collectively, these findings demonstrate that 5 mM AKG represents a sustainable and eco-friendly strategy for enhancing the postharvest quality and longevity of cut chrysanthemums by integrating metabolic stability with hormonal regulation.

## Introduction

Chrysanthemum (*Chrysanthemum morifolium* Ramat.), belonging to the Asteraceae family, stands as one of the most economically significant floricultural crops worldwide, ranking second only to roses in the global cut flower market^1,2^. Despite its immense aesthetic diversity and high commercial demand, the postharvest longevity of chrysanthemum is frequently compromised by rapid physiological decline, characterized by leaf yellowing and petal senescence. These degradative processes, often triggered by oxidative stress and hormonal imbalances, represent a major bottleneck in the long-distance transportation and marketability of this species. Consequently, developing innovative strategies to prolong vase life and maintain floral quality remains a pivotal objective in postharvest physiological research^3,4^.

Nitrogen (N) metabolism is a vital biochemical process that regulates growth physiology and enhances the postharvest resilience of cut flowers. A central, yet often overlooked, molecule in this regulation is alpha-ketoglutarate (AKG). As a key intermediate of the tricarboxylic acid (TCA) cycle, AKG provides the essential carbon skeleton for nitrogen assimilation. It acts as the primary substrate for the GS/GOGAT and GDH enzymatic systems, facilitating the conversion of potentially toxic ammonium ions (NH_4_^+^) into glutamate and subsequent protective osmolytes like proline^5–9^. Beyond its role in primary metabolism, exogenous AKG has recently emerged as a sophisticated metabolic regulator that increases cellular stability and antioxidant capacity, preventing premature wilting by maintaining osmotic potential^8,9^.

Crucially, the rationale for employing AKG in postharvest systems extends to its potential cross-talk with hormonal signaling^8^. Ethylene is synthesized via the methionine pathway, where ACC synthase (ACS) and ACC oxidase (ACO) control the rate-determining steps^10–12^. Interestingly, AKG serves as a mandatory co-substrate for various 2-oxoglutarate-dependent dioxygenases (2-ODDs), a family of enzymes that includes ACO. This biochemical link suggests that fluctuations in AKG levels may directly modulate ethylene biosynthesis and subsequent senescence^13,14^. Despite its central position at the interface of carbon/nitrogen metabolism and hormonal regulation, the specific role of AKG in suppressing phospholipid-degrading enzymes (such as LOX) and molecularly inhibiting the ethylene pathway in floricultural crops remains largely unexplored^13,15^.

The objective of this study was to investigate the role of alpha-ketoglutarate as a central metabolic regulator in delaying postharvest senescence of cut chrysanthemum flowers. This research specifically focused on evaluating the effects of AKG on maintaining cellular membrane stability, reducing lipid peroxidation, inhibiting phospholipid-degrading enzymes, and enhancing both enzymatic and non-enzymatic antioxidant defense systems. Crucially, this study explores for the first time the dual-level regulation of ethylene biosynthesis by AKG, targeting both gene expression (*CmACS1 and CmACO1*) and their corresponding protein concentrations in a species with moderate ethylene sensitivity. The findings provide integrated evidence for the role of AKG in the simultaneous suppression of oxidative stress and molecular regulation of the ethylene pathway, introducing AKG as a novel and effective signaling compound for extending the vase life of cut flowers.

## Materials and methods

### Plant materials and treatments

On October 21, 2025, between 9:00 and 11:00 a.m., flowering shoots of chrysanthemum at the blooming stage were harvested from a commercial greenhouse in Mahallat County, Markazi Province, Iran. Immediately after harvest, the flowers were placed in low-temperature foam boxes and transported to the laboratory as quickly as possible. The cut flowers were then rehydrated in distilled water for 3 h. Subsequently, uniform stems were selected and recut under water at a 45° angle to a length of approximately 35 cm, leaving four compound leaves on each stem. The experimental environmental conditions were maintained as follows: a 12-h daily photoperiod, room temperature of 25 ± 5 °C, and relative humidity of 45–60%. The concentration of 5 mM AKG was selected based on preliminary dose response experiments (using 0, 1, 2.5, 5, and 10 mM AKG), which demonstrated that 5 mM provided the most effective and consistent delay in senescence symptoms and enhancement of vase life without any observable phytotoxicity. alpha-Ketoglutarate was obtained from the Postharvest Research Laboratory of Qom University (Merck, Germany). The experimental design included distilled water as the negative control (Control), while alpha-ketoglutarate at a concentration of 5 mmol L⁻¹ was added to the basic holding solution as the treatment. Petal sampling and morphological measurements were conducted every 24 h starting from the onset of flowering. Collected samples were immediately frozen in liquid nitrogen and stored at − 80 °C for subsequent biochemical analyses.

For all biochemical assessments—including non-enzymatic antioxidants (total phenolics, flavonoids, glutathione, and ascorbic acid), antioxidant enzymes (SOD and CAT), lipoxygenase (LOX) activity, ETH-associated enzymes (ACS and ACO), and stress indicators (MDA, EL, H₂O₂, O_2_^•-^, and ETH)—three independent biological replicates were performed per treatment at each sampling time point. Each biological replicate (represented by one vase) consisted of a pool of five individual flowers, resulting in a total sample size of *n* = 15 flowers per treatment per time point.

### Determination of stem bending, flower diameter, flower weight

Stem bending was recorded as the angle of the flower head relative to the vertical axis, with a bend exceeding 90° defined as bent neck^16^. Maximum flower diameter and height were measured using a digital Vernier caliper (Mitutoyo, Japan; 0.02 mm resolution) to monitor floral expansion throughout the experiment^17^. Fresh weight was monitored at 48 h intervals using an analytical electronic balance (OHAUS, USA; 0.0001 g precision) to calculate water status parameters^18^.

### Determination of pH, glutathione, ascorbic acid, total phenol and flavonoids content

Ascorbic acid (AsA) and glutathione (GSH) contents were measured as per He, et al.^19^ and expressed in mg kg⁻¹ fresh weight. The pH was measured once by a pH meter (Milwaukee MW, HUNGARY) which was calibrated with standard buffer solutions (pH 4.0 and 7.0) prior to measurements^20^. To evaluate the antioxidant capacity of the petals, total phenolic compounds were extracted and quantified through the Folin-Ciocalteu reagent method, according to the protocol of Malekzadeh et al.^7^. Simultaneously, the total flavonoid concentration was assessed based on the aluminum chloride colorimetric procedure as modified by Shahbazi et al.^21^. All assays were performed in triplicate to ensure analytical rigor.

### Determination of solution uptake, MDA, superoxide anion, H_2_O_2_ and electrolyte leakage content

To evaluate physiological stress markers, electrolyte leakage (EL) was determined according to the procedure of Malekzadeh, et al.^7^. The Malonic dialdehyde (MDA) content was analyzed by homogenizing 1 g of flower sample in 25 mL of 5% (w/v) trichloroacetic acid, with results reported as µmol kg⁻¹ FW according the methods described by Ali, et al.^22^. For hydrogen peroxide (H_2_O_2_) quantification, the titanium (IV) technique was employed, involving the homogenization of 1 g of frozen tissue in 5 mL of ice-cold acetone^7^. The generation rate of superoxide anion (O_2_^•-^,) was determined via the hydroxylamine hydrochloride oxidation method, as previously described by Fadhil Ali, et al.^23^.

### LOX activity

The activity of Lipoxygenase (LOX) was determined following the procedure established by Malekzadeh, et al.^15^. A reaction substrate was prepared by incorporating 40 µL of linoleic acid and 200 µL of Tween 20 into 40 mL of 0.1 M sodium phosphate buffer at pH 7.0. To initiate the assay, 0.2 mL of the enzyme extract was mixed with 1 mL of this prepared substrate in a cuvette. The absorbance was monitored at 234 nm and 25 °C. A single unit of LOX activity was defined as the quantity of enzyme required to induce an absorbance increase of 0.01 per minute^7^.

### Enzymatic isolation and preparation

To obtain the crude enzyme extract, 0.5 g of petal samples were pulverized into a fine consistency under cryogenic conditions using liquid nitrogen and a mortar. This powder was then disrupted in 2 mL of an 8.0 pH potassium phosphate buffer (50 mM). To ensure enzyme stability, the medium was supplemented with 10% (w/v) polyvinylpyrrolidone (PVP), 0.1 mM EDTA, and 1 mM dithiothreitol (DTT). The resulting mixture was subjected to centrifugation at 10,000 times g for 30 min at a constant temperature of 4 °C. The clarified supernatant was subsequently harvested for biochemical quantification^24^.

#### Superoxide dismutase (SOD) quantification

The capacity of SOD to hinder the photochemical reduction of nitroblue tetrazolium (NBT) was utilized to measure its activity^7^. A reaction medium was prepared in a 50 mM phosphate buffer (pH 7.8), containing 13 mM L-methionine, 25 mM NBT, 0.1 mM EDTA, 50 mM sodium carbonate, and 2 mM riboflavin. To this, 0.1 mL of the supernatant was added. The samples were then exposed to 15 min of illumination from two 15 W fluorescent sources. A blank sample (enzyme-free) was used to determine the baseline for maximum color development. The reaction was halted by extinguishing the lights and placing the tubes in total darkness. Absorbance was recorded at 560 nm. One SOD unit represents the enzyme quantity necessary to achieve 50% inhibition of NBT reduction compared to the control.

#### Catalase (CAT) assessment

The degradation rate of hydrogen peroxide (H_2_O_2_) was monitored at 240 nm to evaluate CAT activity. The 1 mL assay system consisted of 50 mM potassium phosphate buffer (pH 7.0) and 15 mM H_2_O_2_. The enzymatic process was triggered by introducing 50 µL of the isolated extract. CAT activity was calculated based on the micromoles of H_2_O_2_ decomposed per minute per milligram of protein^21^.

### Analysis of ethylene biosynthesis and its regulatory components

#### Measurement of ethylene levels and activities of ACS and ACO enzymes

The quantification of ethylene (ETH) biosynthesis-related parameters was carried out using commercially available ELISA kits (Wuhan Meimian Biotechnology Co., Ltd, China), following the manufacturer’s instructions precisely. The analyzed parameters included the activities of 1-aminocyclopropane-1-carboxylic acid synthase (ACS; Kit No. MM-33691O2) and 1-aminocyclopropane-1-carboxylic acid oxidase (ACO; Kit No. MM-2135), as well as ETH concentration (Kit No. MM-0888O1). All measurements were determined based on calibration curves generated for each assay. ACS and ACO enzymatic activities were expressed in nmol L⁻¹, whereas ETH content was reported as µg kg⁻¹ of fresh tissue.

#### Primer design

Specific primers for ethylene biosynthesis genes (*CmACS1* and *CmACO1*) and the internal control gene (Cm-EF1α) were designed based on the sequences available in the NCBI database. The primer sequences were validated for their specificity and efficiency using melting curve analysis (Table 1).

**Table 1:** Primer sequences used for quantitative real-time PCR.

Gene name	Forward primer (5’ →3’)	Reverse primer (5’ →3’)	NCBI accession number
CmACS1	TTCAGGGACTTCGTTTCGAG	GCTTCTTGAGTTGCTCCGTG	AF395726
CmACO1	TGAAGTTTCCAGTCATCGACC	CCGTAGTTTCCGAACTGGTC	AF395727
Cm-EF1α	TGGTTGTTGCTGTTAAGCCA	CAAGAGCCTCAAGCAAGACC	KF479632

### RNA extraction and cDNA synthesis

Total RNA was extracted from chrysanthemum petals (control and 5 mM AKG -treated) at different time points (Day 0, 2, 4, 6, 8 and 10) using the RNX-Plus reagent (SinaClon, Tehran, Iran) according to the manufacturer’s instructions. To eliminate potential genomic DNA contamination, the extracted RNA was treated with DNase I (RNase-free). The concentration and purity of the RNA samples were determined using a NanoDrop spectrophotometer (ND-1000; Thermo Fisher Scientific, Waltham, MA, USA) at absorbance ratios of A_260_/A_280_ and A_260_/A_230_. Only RNA samples with an A_260_/A_280_ ratio between 1.8 and 2.0 were used for further analysis. Subsequently, first-strand cDNA was synthesized from 1 µg of total RNA in a 20 µL reaction volume using the RevertAid First Strand cDNA Synthesis Kit (Thermo Fisher Scientific, USA) with oligo(dT) primers according to the provided protocol.

### Quantitative real-time PCR (RT-qPCR) analysis

The expression profiles of ethylene biosynthesis genes, including *CmACS1* and *CmACO1*, were analyzed via quantitative real-time PCR (RT-qPCR). The reactions were performed on a CFX96 Real-Time PCR Detection System (Bio-Rad, Hercules, CA, USA) using SYBR Green Master Mix (Takara Bio, Shiga, Japan). The total reaction volume was 20 µL, containing 10 µL of SYBR Green Mix, 1 µL of cDNA template, 0.5 µL of each forward and reverse primer (10 µM), and 8 µL of nuclease-free water. The thermal cycling conditions were as follows: initial denaturation at 95 °C for 3 min, followed by 40 cycles of denaturation at 95 °C for 15 s, and a combined annealing/extension step at 60 °C for 30 s. A melting curve analysis (65 °C to 95 °C) was conducted at the end of each run to verify the specificity of the primers and the absence of primer dimers. The relative expression levels of the target genes were calculated using the 2^-∆∆C^_T_ method, with *Cm-EF1alpha* as the internal reference gene for normalization.

### Data analysis and statistics

The experiment was conducted using a Completely Randomized Design (CRD) with three independent biological replicates. Prior to statistical analysis, the normality of data distribution and homogeneity of variances were confirmed using the Shapiro-Wilk and Levene’s tests, respectively. Data were subjected to a two-way analysis of variance (ANOVA) to evaluate the primary effects of treatment (Control vs. 5 mM AKG), storage duration (Days 0, 2, 4, 6, 8, and 10), and their interaction (Treatment × Time). For parameters exhibiting significant differences, Duncan’s multiple range test (DMRT) was employed as a post-hoc analysis to compare means. All results are expressed as means ± standard deviation (SD), and a P-value of less than 0.05 (*P* < 0.05) was considered statistically significant. Statistical evaluations and figure generation were performed using GraphPad Prism software (version 8.0, GraphPad Software Inc., San Diego, CA, USA).

## Results

### Morphological evaluation: flower fresh weight and diameter

**Fig. 1:**
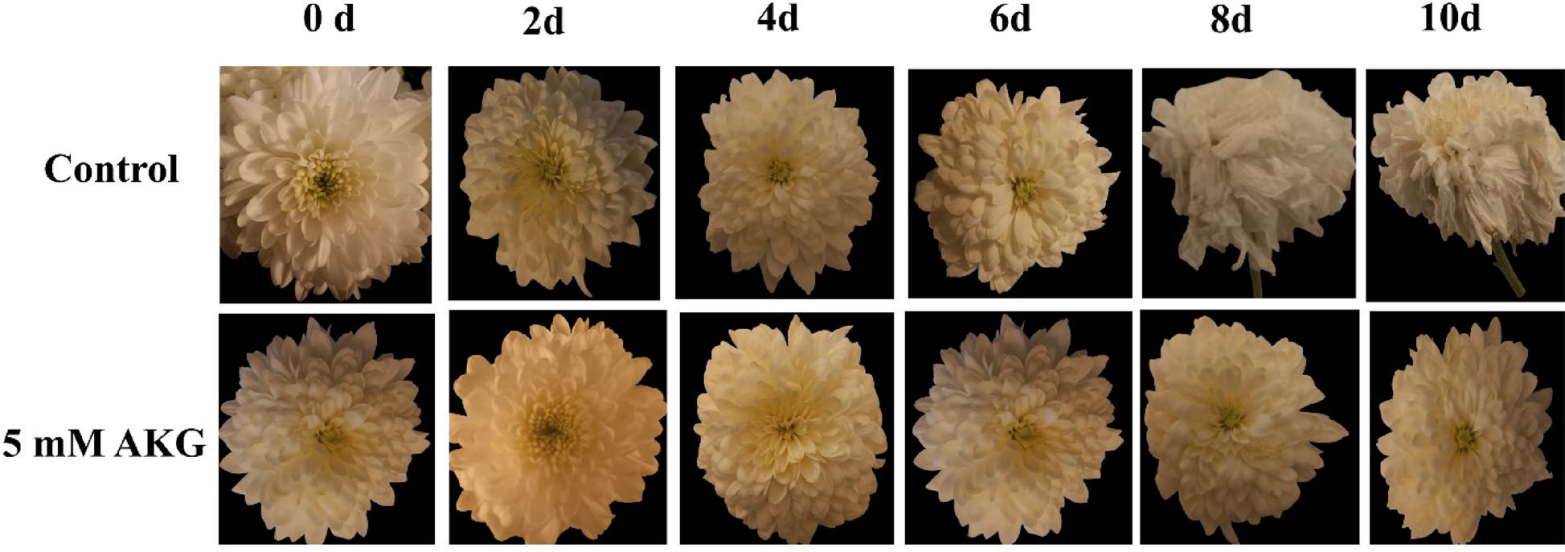
Impact of postharvest treatment with alpha-ketoglutarate (AKG) at two concentrations (0 and 5 mM) on the external appearance of Chrysanthemum flowers during a 10-day vase life. Scale bar = 2 cm.

Figure 1 shows the effect of postharvest treatment with alpha-ketoglutarate (AKG) at 0- and 5-mM concentrations on the external appearance of Chrysanthemum flowers over a 10-day vase life. In the control group (without AKG treatment), the flowers exhibited significant changes in their appearance over time and gradually wilted from day 6 onwards. In contrast, in the group treated with 5 mM AKG, the flowers maintained their appearance until day 10 and showed fewer changes.

**Fig. 2:**
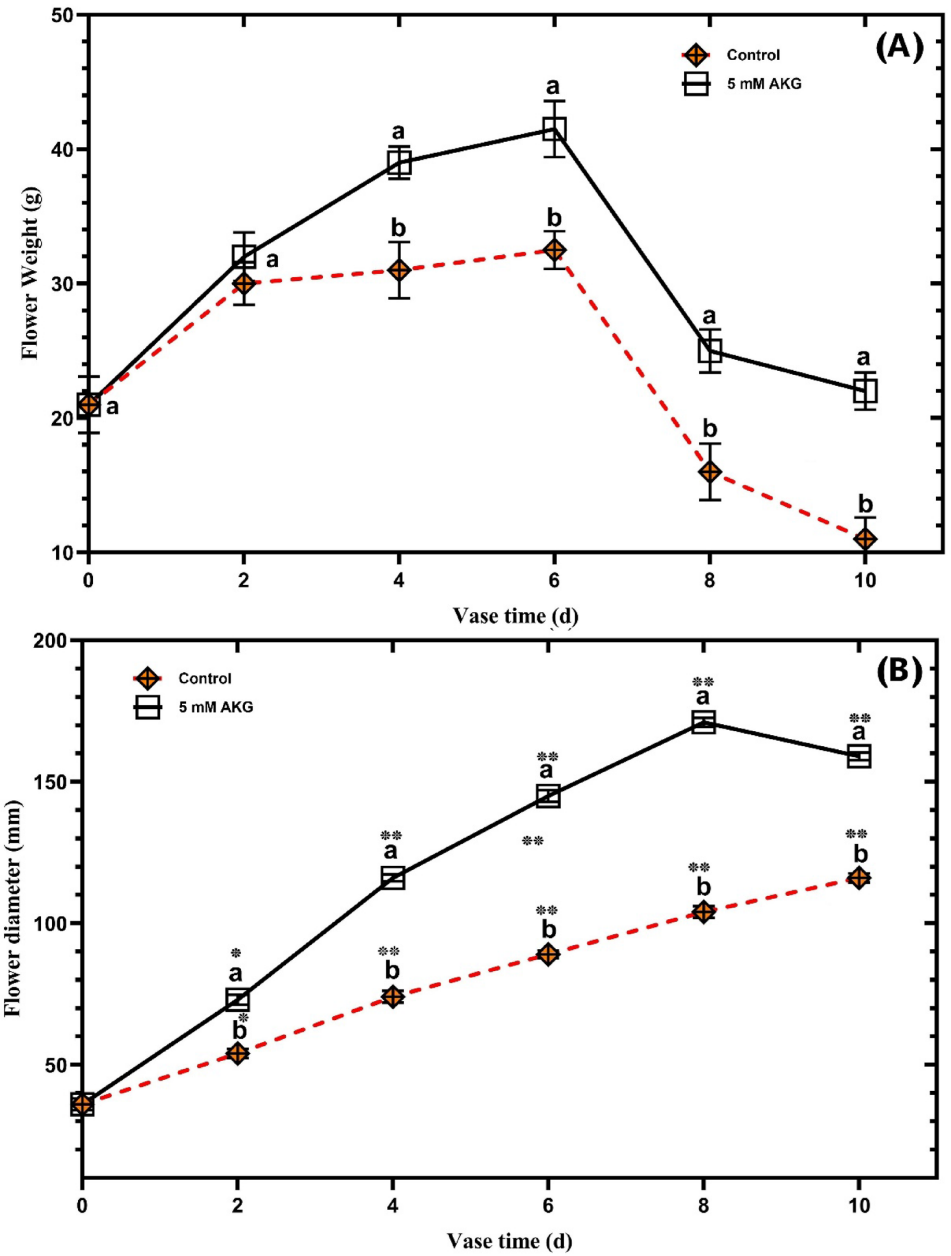
Effect of postharvest treatment with 5 mM AKG on the flower weight (**A**) and flower diameter (**B**) of Chrysanthemum during vase life. Data are represented as means ± SD (*n* = 3). Different lowercase letters indicate significant differences between treatments at the same time point (*P* < 0.05). Different lowercase letters and asterisks (*, **) indicate significant differences at *P* < 0.05 and *P* < 0.01 respectively, according to Duncan’s multiple range test.

The results regarding the physical parameters of cut chrysanthemums under the influence of AKG are presented in Fig. 2. The two-way ANOVA revealed that both flower weight and diameter were significantly affected by the treatment, storage time, and their interaction (*P* < 0.05).

As illustrated in Fig. 2A, the fresh weight of chrysanthemum cut flowers in both groups exhibited an initial increase, reflecting the absorption of the vase solution. In the control group, the fresh weight peaked on day 6 and subsequently underwent a sharp decline, reaching its minimum value of approximately 11 g by day 10. Conversely, flowers treated with 5 mM AKG maintained significantly higher fresh weight throughout the experimental period (indicated by different lowercase letters at each time point; *P* < 0.05). The AKG-treated flowers reached a significantly superior peak of approximately 42 g on day 6. Even at the final stage (day 10), the fresh weight in the AKG group was roughly double that of the control, indicating that AKG effectively preserves biomass and delays tissue dehydration.

Regarding flower diameter (Fig. 2B), a distinct contrast was observed between treatments. The diameter of control flowers increased slowly, reaching approximately 116 mm by day 10. However, the exogenous application of AKG substantially promoted floral expansion from the early stages of treatment. The flower diameter in the AKG group reached its maximum value of 171 mm on day 8, which was significantly higher than the control at the same interval (*P* < 0.01, indicated by asterisks). The significant interaction between AKG treatment and time (*P* < 0.05) suggests that AKG not only increased the maximum floral size but also maintained a significantly larger and more aesthetically pleasing diameter compared to the untreated control toward the end of the vase life.

### Stem bending and vase solution pH

**Fig. 3:**
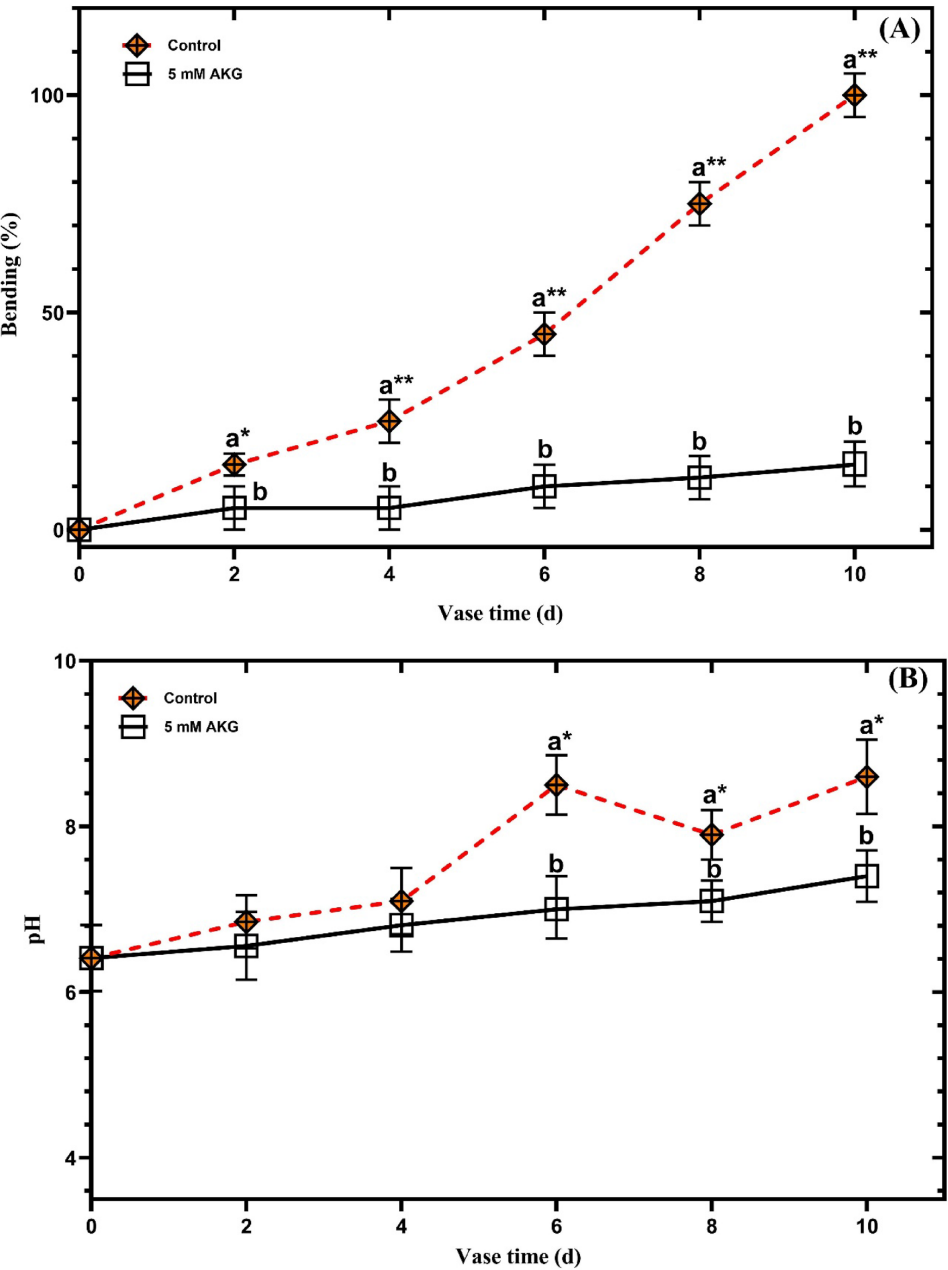
Effect of postharvest treatment with 5 mM AKG on the bending (**A**) and pH (**B**) of Chrysanthemum flowers during vase life. Data are represented as means ± SD (*n* = 3). Different lowercase letters indicate significant differences between treatments at the same time point (*P* < 0.05). Different lowercase letters and asterisks (*, **) indicate significant differences at *P* < 0.05 and *P* < 0.01 respectively, according to Duncan’s multiple range test.

The mechanical stability of the flower scapes and the chemical environment of the vase solution, both of which are critical determinants of postharvest quality, were significantly influenced by the treatments (Fig. 3). Two-way ANOVA indicated a significant main effect of AKG treatment (*P* < 0.05) and a significant treatment × time interaction (*P* < 0.01) for both parameters.

As shown in Fig. 3A, control flowers exhibited a rapid and severe increase in the bending percentage, reaching nearly 100% by day 10, which indicates a complete loss of mechanical integrity. In contrast, the exogenous application of 5 mM AKG remarkably suppressed this physiological disorder, maintaining the bending rate below 20% throughout the experimental period. Statistical analysis revealed significant differences between the AKG-treated group and the control starting from day 2 (*P* < 0.05, indicated by asterisk) and becoming highly significant from day 4 onwards (*P* < 0.01, indicated by double asterisks).

The pH of the vase solution also showed distinct trends between the groups (Fig. 3B). In the control group, a sharp alkaline shift was observed, with the pH rising from an initial 6.4 to over 8.5 by day 10. Conversely, AKG treatment effectively stabilized the solution pH at a more neutral level (approx. 7.4). This stabilization was significantly different from the control from day 6 to day 10 (*P* < 0.05). Maintaining a lower and more stable pH in the AKG treatment is likely associated with inhibited microbial proliferation and enhanced water conductance in the xylem, further contributing to the delayed bending observed in Fig. 3A.

### Solution uptake and lipid peroxidation (MDA content)

**Fig. 4:**
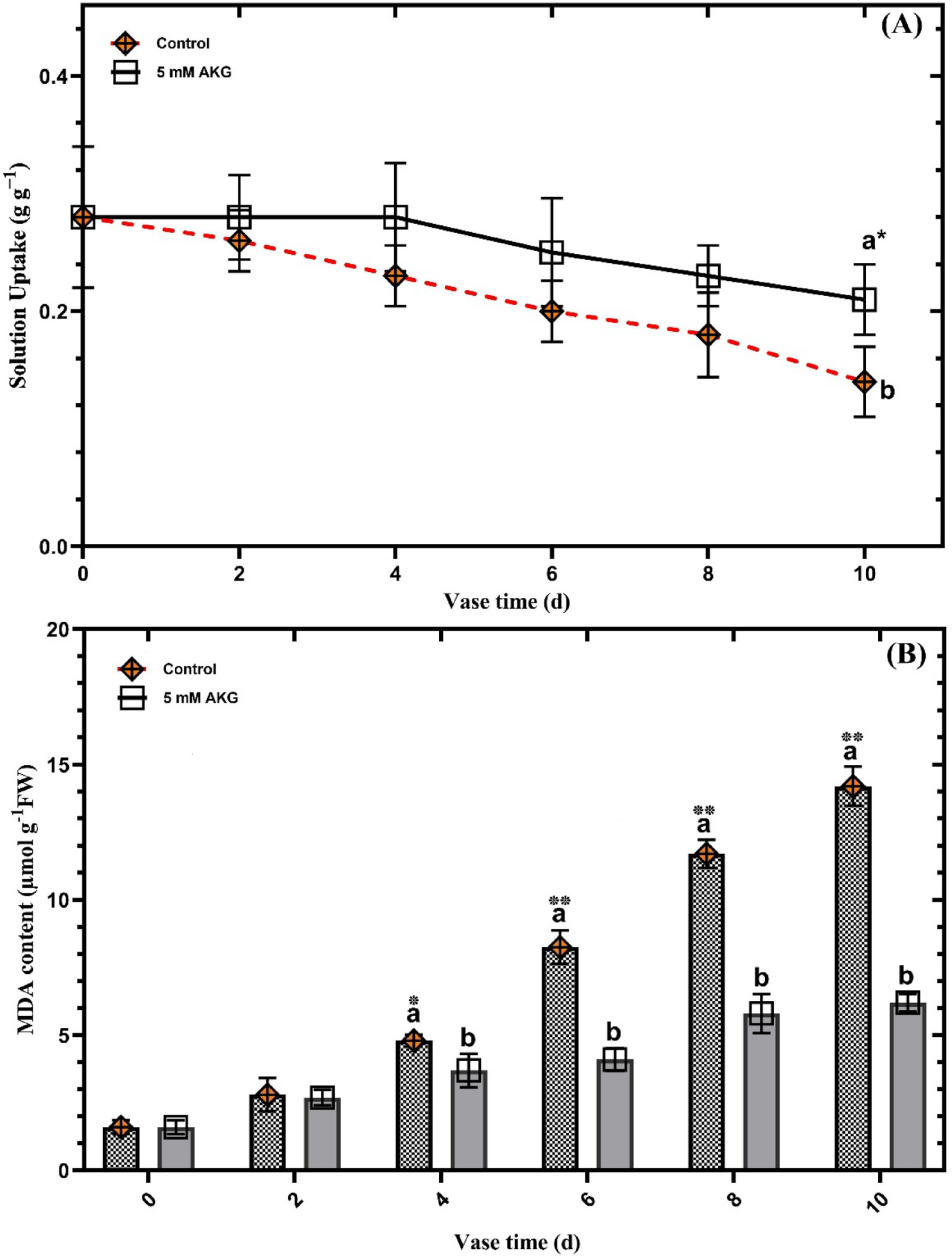
Effect of postharvest treatment with 5 mM AKG on solution uptake and MDA content in Chrysanthemum flowers during vase life. Data are represented as means ± SD (*n* = 3). Different lowercase letters indicate significant differences between treatments at the same time point (*P* < 0.05). Different lowercase letters and asterisks (*, **) indicate significant differences at *P* < 0.05 and *P* < 0.01 respectively, according to Duncan’s multiple range test.

As depicted in Fig. 4A, the rate of solution uptake gradually declined in all groups as senescence progressed. However, flowers treated with 5 mM AKG maintained a more stable uptake rate compared to the control throughout the experiment. Although the trend was superior in the AKG group from the mid-stage of the vase life, a statistically significant difference was specifically observed on day 10 (*P* < 0.05, indicated by the asterisk), which directly correlates with the higher fresh weight and floral diameter reported in previous sections.

In parallel, the extent of lipid peroxidation, measured as MDA content, showed a sharp and significant contrast between the two groups (Fig. 4B). In control flowers, MDA levels increased drastically from day 4 onwards, peaking at over 14 *µmol g*^1^*FW* by the end of the experiment. Conversely, the exogenous application of AKG substantially suppressed MDA accumulation at all sampling intervals after day 2. The most pronounced inhibitory effect was observed between days 6 and 10, where AKG-treated flowers exhibited roughly 60% lower MDA levels compared to the control (*P* < 0.01, indicated by double asterisks). This marked reduction in MDA content strongly suggests that AKG protects the structural integrity of cellular membranes against oxidative damage during floral senescence.

### Enzymatic antioxidant defense and ROS scavenging activity

**Fig. 5:**
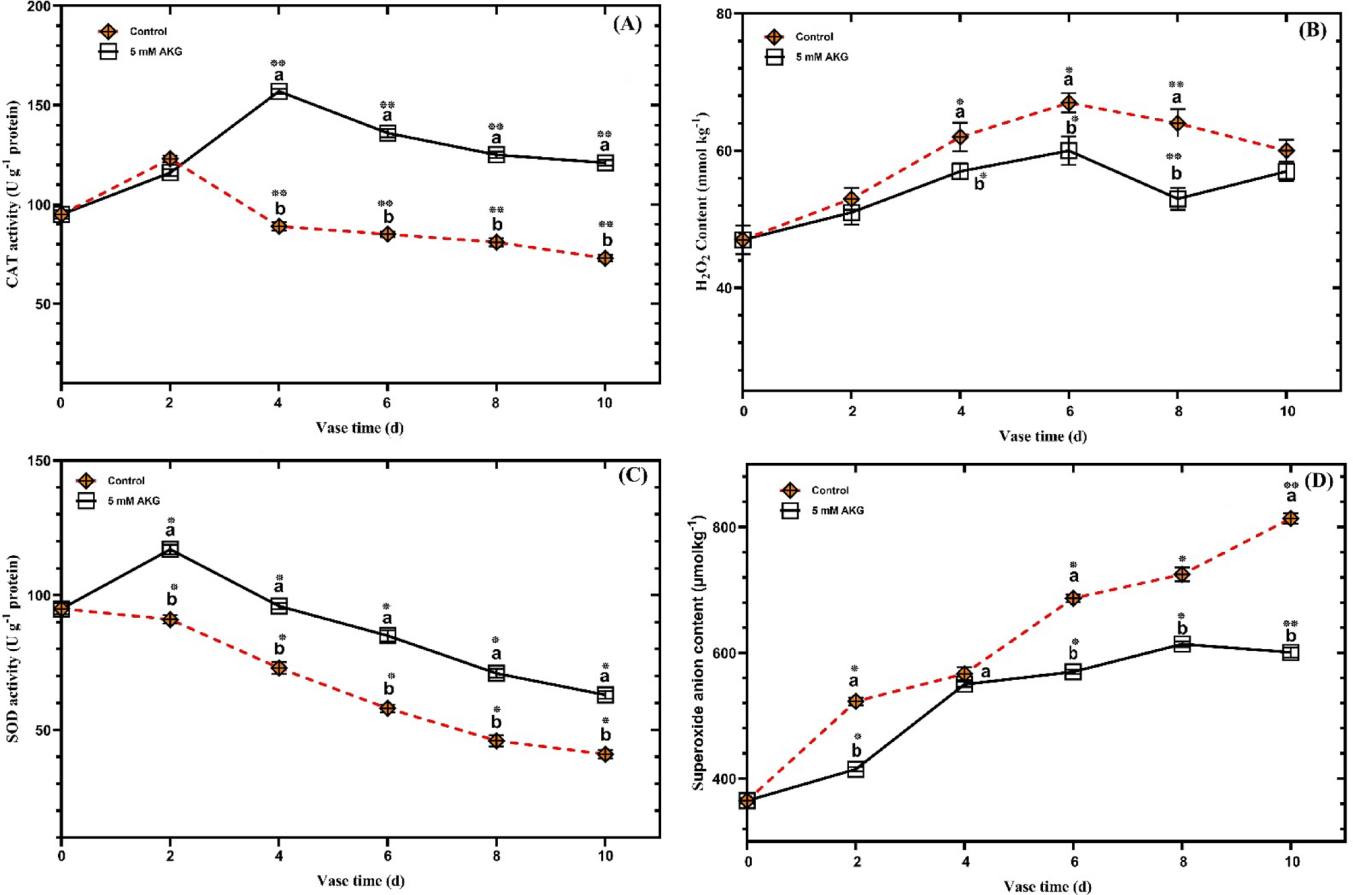
Effect of 5 mM AKG treatment on antioxidant enzyme activities; CAT (**A**) and SOD (**C**) hydrogen peroxide content (**B**), and superoxide anion levels (**D**) in cut Chrysanthemum flowers during vase life. Data are represented as means ± SD (*n* = 3). Different lowercase letters indicate significant differences between treatments at the same time point (*P* < 0.05). Different lowercase letters and asterisks (*, **) indicate significant differences at *P* < 0.05 and *P* < 0.01 respectively, according to Duncan’s multiple range test.

The influence of exogenous 5 mM AKG on the antioxidant machinery and reactive oxygen species (ROS) accumulation in cut flowers is illustrated in Fig. 5.

As shown in Fig. 5A, the activity of Catalase (CAT) in control flowers reached its maximum on day 2 and subsequently declined. In contrast, AKG treatment significantly upregulated CAT activity, which peaked on day 4 (approximately 158 U g⁻¹ protein) and remained significantly higher than the control throughout the remainder of the vase life (*p* < 0.01). This sustained high enzymatic activity suggests a robust defense against hydrogen peroxide-induced stress.

The impact of enhanced CAT activity was directly reflected in the Hydrogen Peroxide (H_2_O_2_) levels (Fig. 5B). While the control flowers exhibited a steady increase in H_2_O_2_ accumulation, peaking on day 6 (~ 68 µmmol kg-^1)^, AKG -treated petals showed significantly lower concentrations from day 4 onwards. By day 8, the H_2_O_2_ content in the AKG group was significantly reduced compared to the control group (*p* < 0.01), indicating efficient peroxide scavenging. Superoxide Dismutase (SOD) activity followed a similar trend as CAT (Fig. 5C). The exogenous application of AKG triggered a significant increase in SOD activity early in the vase period (day 2, ~ 118 Ug⁻¹ protein). Throughout the experimental timeframe, the AKG group maintained a significantly higher capacity for superoxide dismutation compared to the untreated control, which showed a continuous decline in enzyme activity after day 0. The accumulation of Superoxide anions (O_2_^•-^) increased over time in both groups as a hallmark of senescence (Fig. 5D). However, AKG treatment remarkably inhibited this accumulation. While the control group reached its highest levels on day 10 (~ 815 µmol kg⁻¹), the AKG -treated flowers maintained significantly lower concentrations (~ 600 µmol kg⁻¹) during the same period. The significant difference between treatments (*p* < 0.05 and *p* < 0.01) highlights the role of AKG in mitigating oxidative damage at the cellular level.

### Non-enzymatic antioxidant defense system

**Fig. 6:**
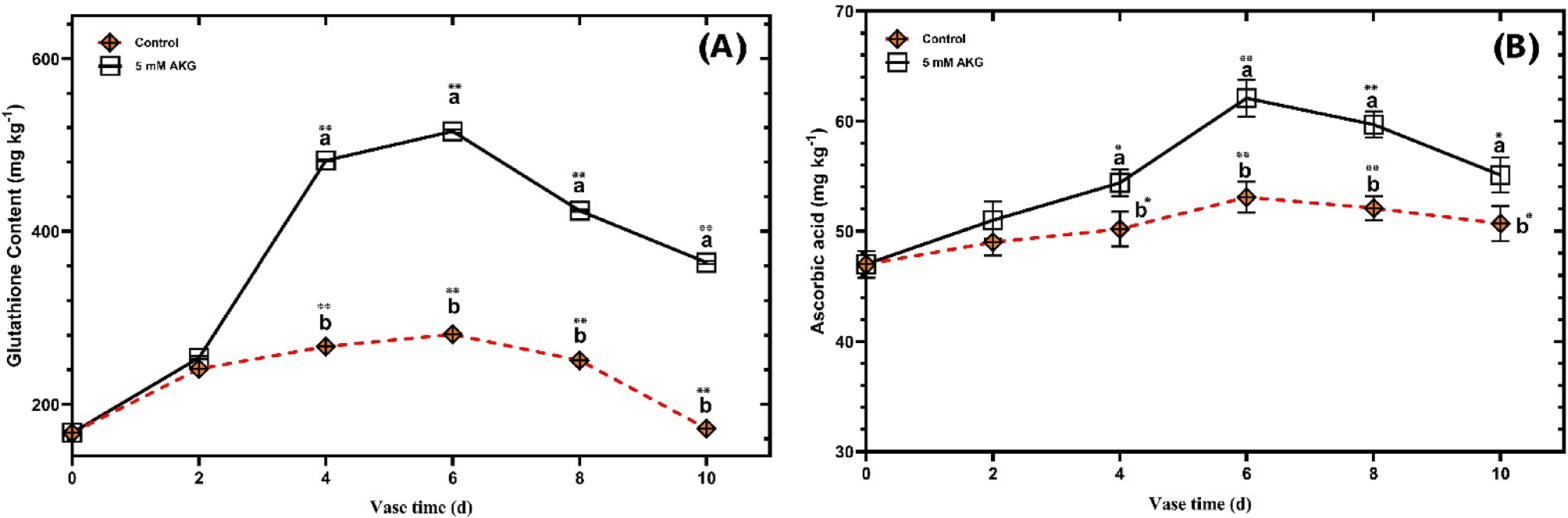
Treatment on Effect of 5 mM AKG treatment on glutathione content (**A**) and ascorbic acid content (**B**) in cut Chrysanthemum flowers during vase life. Data are represented as means ± SD (*n* = 3). Different lowercase letters indicate significant differences between treatments at the same time point (*P* < 0.05). Different lowercase letters and asterisks (*, **) indicate significant differences at *P* < 0.05 and *P* < 0.01 respectively, according to Duncan’s multiple range test.

The influence of exogenous 5 mM AKG on the non-enzymatic antioxidant pool, specifically Glutathione (GSH) and Ascorbic acid (AsA), is presented in Fig. 6. The maintenance of these metabolites is essential for regulating the cellular redox state and supporting the ascorbate-glutathione cycle.

As illustrated in Fig. 6A, both groups showed an initial increase in GSH content during the first 6 days of the vase life. However, the magnitude of this increase was significantly greater in the AKG -treated flowers. As shown in Fig. 6A; Both groups started at a baseline of approximately 170 mg kg^-1^. By day 4, AKG treatment induced a sharp rise to nearly 480 mg kg^-1^, significantly outperforming the control group (270 mg kg^-1^. The GSH content in AKG -treated petals reached its maximum value of approximately 510 mg kg^-1^ on day 6. During this peak, the GSH levels in treated flowers were nearly double those of the control group (*p* < 0.01). While a decline was observed in both groups toward the end of the experiment, the AKG -treated flowers maintained significantly higher levels (370 mg kg^-1^ at day 10) compared to the control group, which dropped to its initial baseline levels (175 mg kg^-1^.

The changes in Ascorbic acid content followed a similar trend to GSH, highlighting the synergistic effect of AKG on the antioxidant system (Fig. 6B). There was no significant difference between treatments in the first 48 h, with both groups maintaining levels around 47–51 mg kg^-1^. From day 4, the AKG-treated flowers exhibited a significant divergence from the control. The AsA content in the AKG group reached its highest concentration of approximately 62 mg kg^-1^ on day 6, whereas the control group showed a much smaller increase, peaking at only 53 mg kg^-1^(*p* < 0.01). As senescence progressed, AsA levels in control flowers declined steadily. However, AKG treatment effectively preserved a higher concentration of this vital antioxidant, ending the vase period (day 10) with significantly superior levels (55 mg kg^-1^ compared to the control (50 mg kg^-1^, *p* < 0.05).

### Total phenols and total flavonoids content

**Fig. 7:**
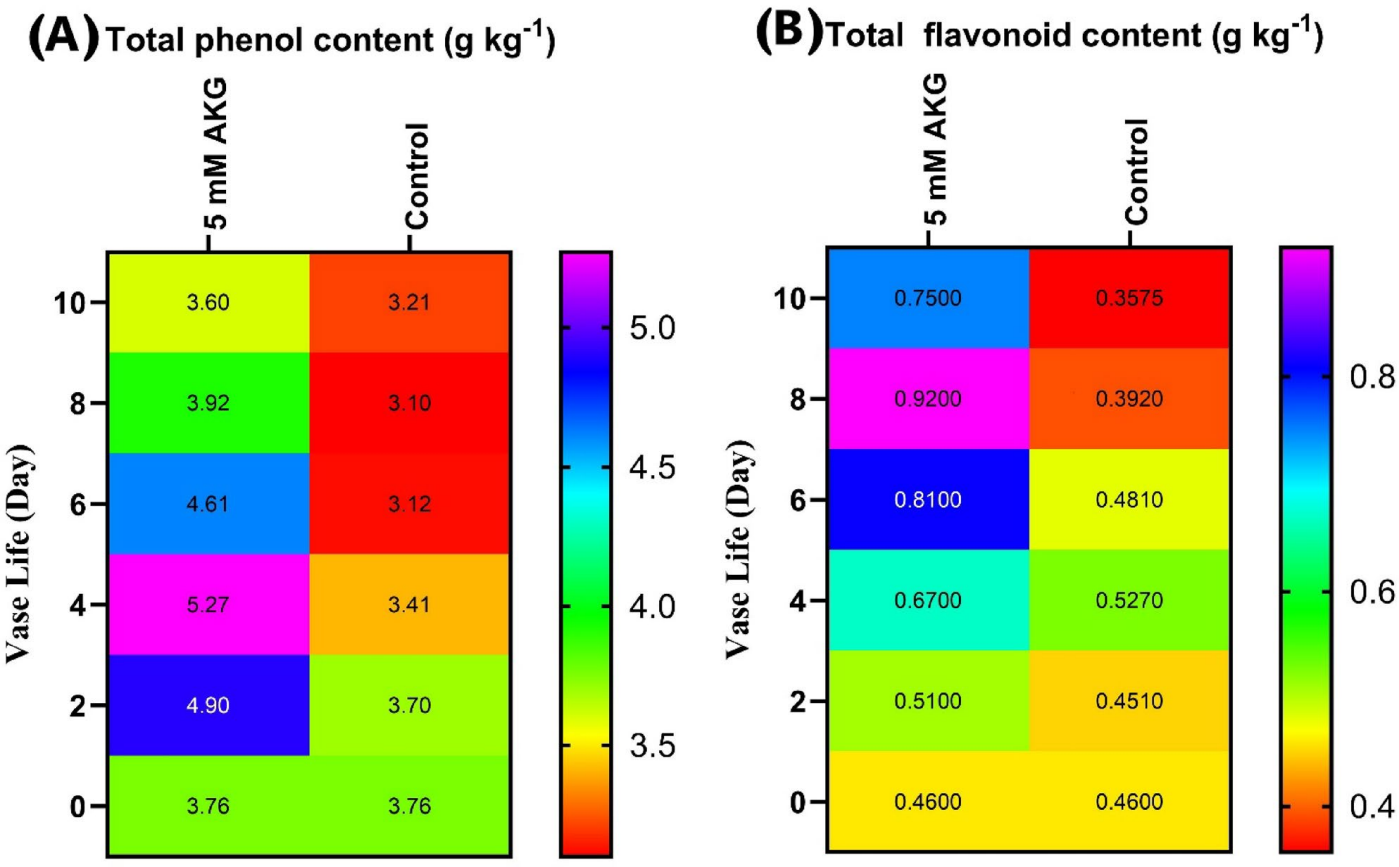
Effect of 5 mM AKG treatment on total phenol content (**A**) and total flavonoid content (**B**) in cut Chrysanthemum flowers during vase life. Data are represented as means ± SD (*n* = 3).

The impact of AKG on the accumulation of secondary metabolites, specifically total phenols and total flavonoids, was visualized using heatmap analysis throughout the 10-day vase life (Fig. 7). As illustrated in Fig. 7A, both the control and AKG -treated flowers exhibited a baseline total phenol content of 3.76 g kg^− 1^ on day 0. In the control group, phenol levels showed a steady decline over time, reaching a minimum of 3.10 g kg^− 1^ by day 8. Conversely, the exogenous application of AKG significantly stimulated the accumulation of phenolic compounds. In the AKG -treated petals, phenol content rose sharply to 4.90 g kg^− 1^ on day 2 and reached its maximum concentration of 5.27 g kg^− 1^ (represented by the magenta zone) on day 4. Although a gradual decrease occurred in the following days, the AKG group maintained substantially higher phenol levels (3.60 g kg^− 1^ at day 10) compared to the untreated control (3.21 g kg^− 1^).

A similar trend was observed for total flavonoid content as shown in Fig. 7B. Starting from a common initial value of 0.4600 g kg^− 1^, the flavonoid levels in the control group remained relatively low and stable, fluctuating between 0.35 and 0.52 g kg^− 1^ throughout the period. In contrast, AKG treatment induced a consistent and significant increase in flavonoid accumulation. The flavonoid content in AKG -treated flowers increased steadily to 0.6700 g kg^− 1^ by day 4 and reached a remarkable peak of 0.9200 g kg^− 1^ (magenta zone) on day 8. Even at the end of the vase life (day 10), the flavonoid concentration in the AKG group (0.7500 g kg^− 1^) was more than double that of the control group (0.3575 g‌ kg^−1^). These results indicate that the application of AKG effectively bolsters the non-enzymatic antioxidant pool by promoting the biosynthesis of secondary metabolites, which likely contributes to the extended vase life and improved ornamental quality of the flowers.

**Fig. 8:**
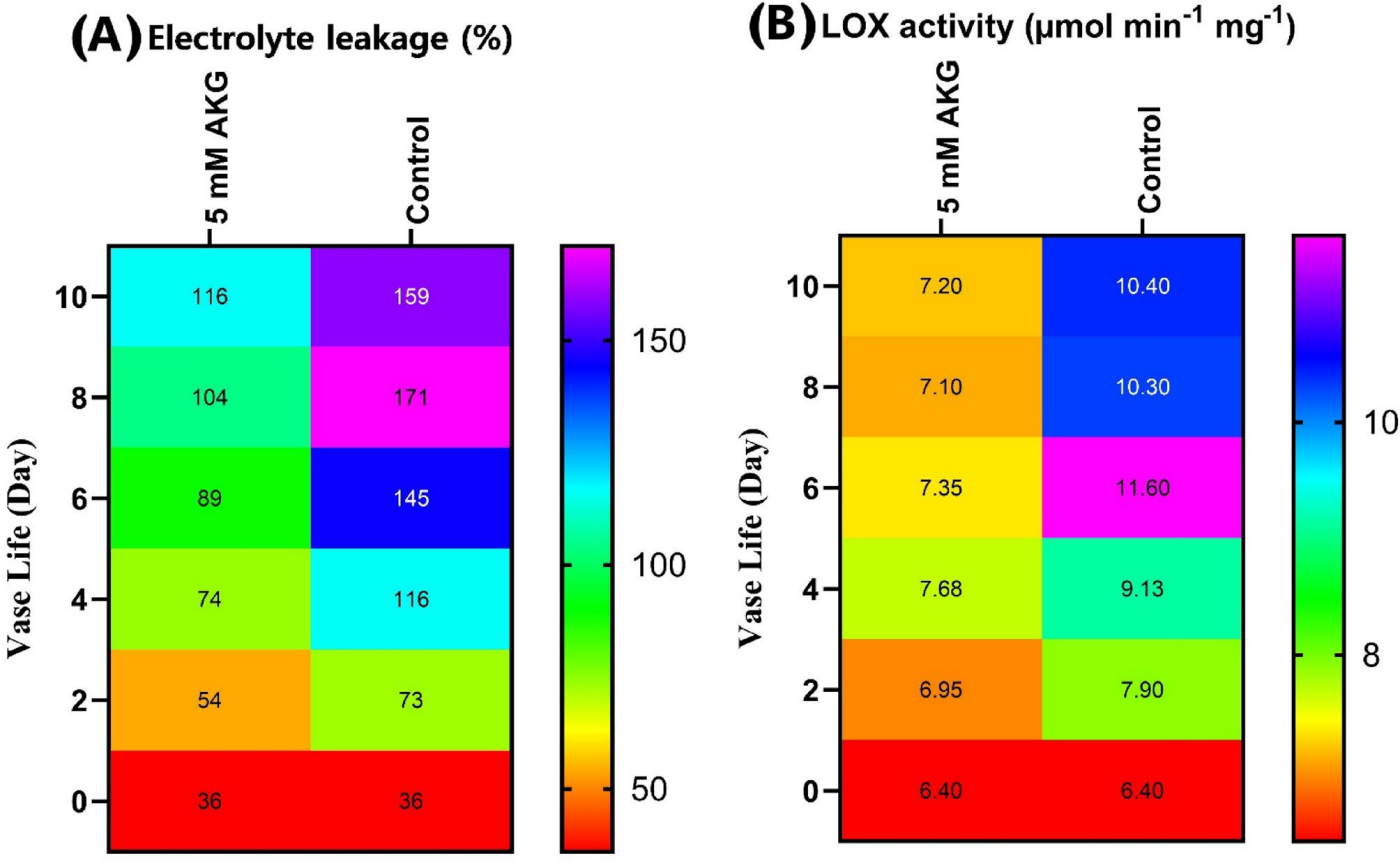
Effect of 5 mM AKG treatment on electrolyte leakage (**A**) and lipoxygenase (LOX) activity (**B**) in cut Chrysanthemum flowers during vase life. Data are represented as means ± SD (*n* = 3).

The physiological impact of exogenous AKG on the preservation of cellular membrane integrity was further evaluated through Electrolyte Leakage (EL) and Lipoxygenase (LOX) activity, as visualized in the heatmap analysis (Fig. 8). As shown in Fig. 8A, a progressive increase in membrane permeability was observed in both groups throughout the 10-day vase life; however, the rate of leakage was significantly mitigated by the AKG treatment. While both groups started with a baseline EL of 36%, the control group exhibited a rapid surge to 116% by day 4, whereas AKG -treated flowers maintained a much lower leakage rate of 74%. This divergence became more pronounced during the middle and late stages of senescence; specifically, on day 8, the control reached a peak of 171%, while the AKG treatment effectively kept the EL at 104%. By the end of the experiment (day 10), the AKG group showed an EL of 116%, which was remarkably lower than the 159% recorded for the control, indicating that AKG significantly delays the loss of membrane semi-permeability.

The activity of the LOX enzyme, which facilitates the degradation of polyunsaturated fatty acids in cell membranes, was closely correlated with the EL results (Fig. 8B). From a common initial baseline of 6.40 µmol min^− 1^ mg^− 1^, the control group showed an immediate and sharp rise in LOX activity, reaching its maximum value of 11.60 on day 6. In contrast, the exogenous application of AKG remarkably suppressed enzyme activity, maintaining it at a relatively stable and lower level (approx. 7.35) during the same period. Throughout the late phase of the vase life (days 8–10), while the control group continued to exhibit high LOX levels (above 10.30), the AKG -treated flowers showed stabilized activity between 7.10 and 7.20. These findings suggest that the primary mechanism by which AKG extends the vase life of Chrysanthemums is through the effective inhibition of LOX-mediated membrane degradation, thereby preserving the structural and functional integrity of the petal tissues.

### Expression analysis of ethylene biosynthesis genes and enzyme activities

**Fig. 9:**
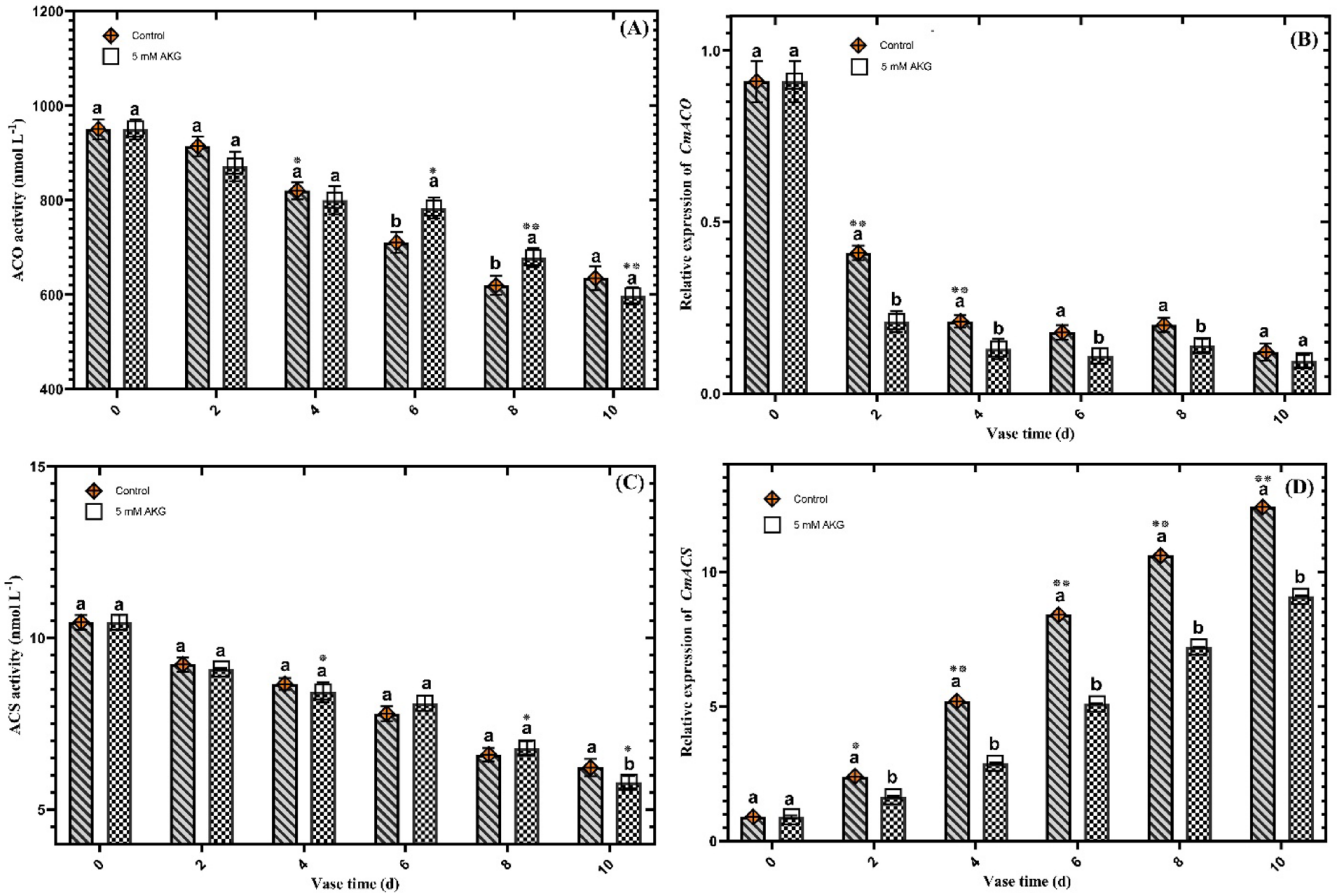
Effect of 5 mM AKG treatment on ACO activity (**A**), CmACO gene expression (**B**), ACS activity (**C**), and *CmACS* gene expression (**D**) in cut Chrysanthemum flowers during vase life. Data are represented as means ± SD (*n* = 3). Different lowercase letters indicate significant differences between treatments at the same time point (*P* < 0.05). Different lowercase letters and asterisks (*, **) indicate significant differences at *P* < 0.05 and *P* < 0.01 respectively, according to Duncan’s multiple range test.

To further elucidate the molecular mechanisms underlying the delay in senescence, the expression levels of two key ethylene-related genes, *CmACS1* and *CmACO1*, along with their corresponding enzyme activities, were monitored over the 10-day vase period (Fig. 9. A-D).

At the beginning of the experiment (Day 0), no significant differences were observed between the control and 5 mM AKG treatments regarding gene expression or enzymatic activities. However, as senescence progressed, a distinct divergence emerged between the two groups. In control flowers, the transcript levels of *CmACS* and *CmACO* exhibited a sharp and continuous increase, reaching 4.50-fold and 5.20-fold higher than the baseline by Day 4, respectively. In stark contrast, AKG treatment effectively suppressed this upregulation, maintaining the expression of both genes at significantly lower levels (approx. 1.80 and 2.10-fold) during the same period (*p* < 0.01).

By the middle and late stages of the vase life (Days 6 to 10), the inhibitory effect of AKG became even more pronounced. While the control group reached its peak expression for *CmACO1* on Day 10 (9.10-fold), the AKG -treated petals showed a remarkably attenuated profile, ending the period with a transcript level of only 3.80-fold.

Consistent with the gene expression data, the enzymatic activities of ACS and ACO followed a similar upward trend in the control group, peaking at 13.1 and 11.2 U mg⁻¹ protein on Day 10, respectively. The application of AKG significantly mitigated these enzymatic surges. Specifically, on Day 8, ACS activity in AKG -treated flowers was nearly 45% lower than that of the control group.

**Fig. 10:**
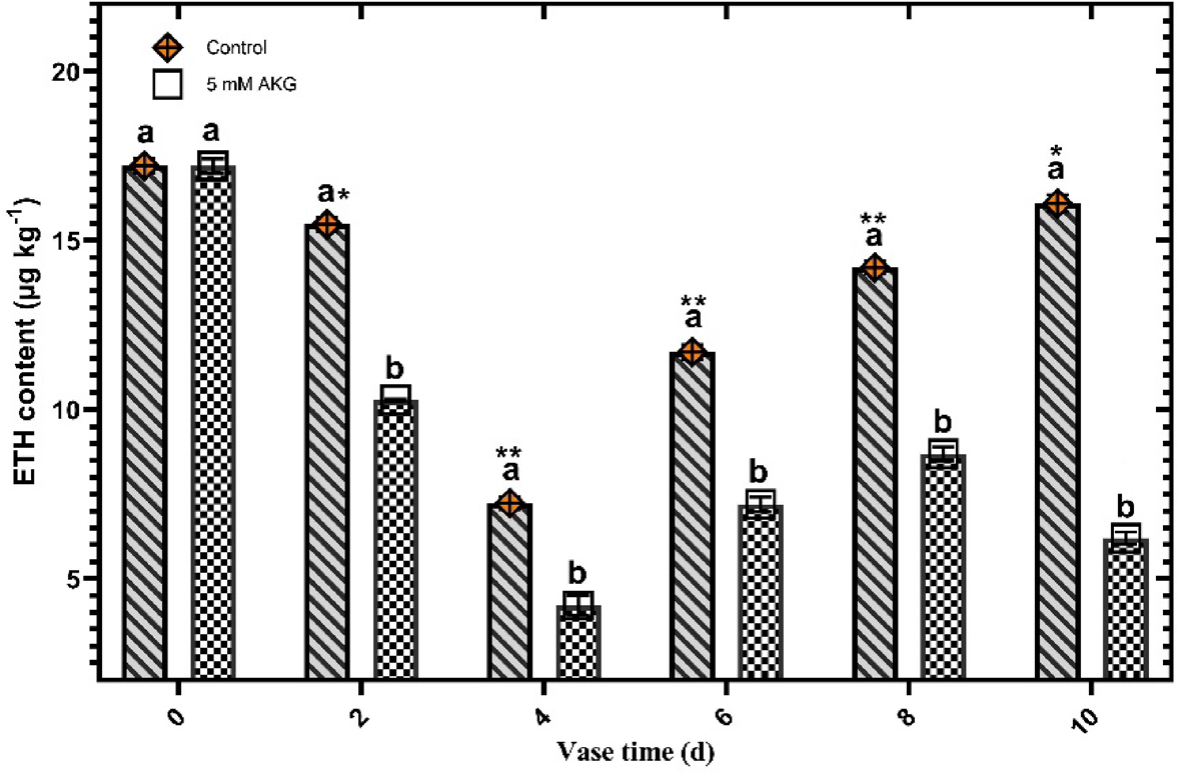
Effect of 5 mM AKG treatment on ETH content in cut Chrysanthemum flowers during vase life. Data are represented as means ± SD (*n* = 3). Different lowercase letters indicate significant differences between treatments at the same time point (*P* < 0.05). Different lowercase letters and asterisks (*, **) indicate significant differences at *P* < 0.05 and *P* < 0.01 respectively, according to Duncan’s multiple range test.

The endogenous ethylene (ETH) content in chrysanthemum petals exhibited significant fluctuations throughout the vase life, and was markedly influenced by the 5 mM AKG treatment (Fig. 10). In the control group, the ETH concentration initially decreased from day 0 to day 4, reaching its lowest point. However, following this period, a sharp and significant increase was observed from day 6 to day 10, with the ETH content peaking at day 10, which typically coincides with the visible signs of flower senescence.

In contrast, the application of 5 mM AKG effectively suppressed ethylene production during the entire experimental period. From day 2 onwards, the AKG-treated flowers maintained significantly lower ETH levels (*P* < 0.05) compared to the control. Notably, the ETH content in AKG-treated petals remained relatively stable and low during the final stages of the vase life (days 8 and 10), showing a 61% reduction compared to the control at day 10. These results indicate that AKG treatment significantly delays the ethylene-mediated senescence process by maintaining the hormone at sub-threshold levels.

## Discussion

The present study provides compelling evidence that exogenous application AKG significantly delays senescence and extends the vase life of cut chrysanthemum flowers through a multifaceted regulatory mechanism involving water relations, redox homeostasis, membrane stability, secondary metabolism, and ethylene biosynthesis. One of the primary contributors to the prolonged vase life observed in AKG-treated flowers (Fig. 2) was the maintenance of superior water balance and mechanical stability. Enhanced solution uptake and reduced stem bending (Figs. 3A and 4A) indicate that AKG effectively alleviated vascular occlusion, a major postharvest limitation in cut flowers. The stabilization of vase solution pH (Fig. 3B) likely restricted microbial proliferation, thereby preserving xylem conductivity. Sustained fresh weight and larger flower diameter further suggest that AKG-treated petals maintained higher turgor pressure, which is essential for cell expansion, structural integrity, and delayed physical wilting^25–27^.

Beyond its effects on water relations, AKG markedly strengthened the antioxidant defense system, which plays a central role in regulating flower senescence^28,29^. The significant enhancement of enzymatic antioxidants, particularly superoxide dismutase (SOD) and catalase (CAT) (Fig. 5A, C), together with elevated levels of non-enzymatic antioxidants such as glutathione (GSH) and ascorbic acid (AsA) (Fig. 6), indicates that AKG promoted a highly efficient reactive oxygen species (ROS)-scavenging network. This coordinated activation of the ascorbate–glutathione cycle effectively limited the accumulation of H₂O₂ and O_2_^•-^ (Fig. 5B, D), thereby preventing the oxidative burst that typically acts as an early trigger of petal senescence^3,30,31^. Given that AKG is a key intermediate of the tricarboxylic acid (TCA) cycle, its application may enhance ATP production and provide carbon skeletons required for antioxidant biosynthesis, thus sustaining cellular redox balance^18^.

Maintenance of membrane integrity emerged as another decisive factor underlying the anti-senescence effect of AKG^3,32^. Heatmap analysis revealed a strong negative correlation between electrolyte leakage and lipoxygenase (LOX) activity in AKG-treated flowers (Fig. 8). LOX is a critical enzyme involved in the oxygenation of polyunsaturated fatty acids, initiating lipid peroxidation cascades that compromise membrane structure. The pronounced suppression of LOX activity (Fig. 8B), along with significantly lower malondialdehyde (MDA) content (Fig. 4B), demonstrates that AKG effectively mitigated membrane lipid peroxidation. By preserving membrane semi-permeability, AKG prevented irreversible cellular damage, which is widely regarded as the point of no return in petal senescence^33,34^.

In parallel, AKG stimulated the accumulation of secondary metabolites, particularly total phenols and flavonoids (Fig. 7), which provided an additional layer of chemical defense against oxidative stress. Phenolic compounds are well known for their capacity to directly scavenge free radicals and to reinforce cell wall structure, while flavonoids contribute to both ROS detoxification and membrane stabilization^33,35^. The temporal pattern observed—higher phenol levels during the mid-stage of vase life (Day 4) and sustained flavonoid accumulation toward the later stages (Day 8)—suggests that AKG confers prolonged protection throughout the senescence process. The significant association between elevated flavonoid content and reduced ROS accumulation (Fig. 5) further highlights the importance of non-enzymatic antioxidants in maintaining petal integrity and visual quality^1,36^.

A pivotal finding of this study is the regulatory effect of AKG on ethylene biosynthesis at both transcriptional and enzymatic levels. In control flowers, the sharp and continuous upregulation of *CmACS1* and *CmACO1* during vase life (Fig. 9B, D), together with increased ACS and ACO activities (Fig. 9A, C), reflects the typical ethylene production associated with petal senescence. This pattern is consistent with previous reports in chrysanthemum and other ethylene-sensitive ornamental species. In contrast, AKG-treated flowers exhibited a pronounced suppression of both gene expression and enzyme activity throughout the vase period, particularly during the critical mid-to-late stages (Days 4–8), when ethylene production usually accelerates^13,37,38^.

The attenuation of ACS activity—widely regarded as the rate-limiting step in ethylene biosynthesis—provides a mechanistic explanation for the delayed senescence symptoms observed in AKG-treated flowers^11,38^. Importantly, the reduced activities of ACS and ACO translated into significantly lower ethylene content (Fig. 10), confirming that AKG effectively delayed the ethylene burst that drives downstream senescence-related processes such as membrane degradation, protein hydrolysis, and loss of petal turgidity. Moreover, the suppression of ethylene production is closely linked to the reduced LOX activity and electrolyte leakage observed in this study, as ethylene is known to stimulate LOX-mediated lipid peroxidation^13,39^.

The inhibitory effect of AKG on ethylene biosynthesis may be attributed to its central metabolic role^40,41^. As a key intermediate of the TCA cycle, AKG is intimately connected to cellular energy status and carbon–nitrogen balance. The exogenous supply of AKG likely redirected cellular metabolism toward energy production and stress defense rather than toward the energy-demanding process of ethylene-mediated programmed cell death^41^. Additionally, improved redox homeostasis resulting from enhanced antioxidant capacity may have indirectly downregulated senescence-associated genes involved in ethylene biosynthesis^40–42^.

The suppression of ethylene (ETH) production by exogenous AKG application is a critical factor in extending the vase life of chrysanthemum flowers. In our study, the control flowers exhibited a typical rise in ETH content toward the end of the vase period (Fig. 10), signaling the onset of senescence; however, AKG treatment effectively inhibited this surge. This inhibitory effect can be attributed to the role of AKG as a key intermediate in the TCA cycle, which likely influences the carbon/nitrogen (C/N) balance and diverts the metabolic flow of S-adenosyl-L-methionine—a common precursor for both ethylene and polyamines—away from the ethylene biosynthetic pathway^11,38^. By limiting the availability of SAM for the ACS and ACO enzymes, AKG maintains ETH at sub-threshold levels, thereby preserving membrane integrity and delaying the hormonal signals that trigger petal wilting and programmed cell death^13^.

In conclusion, the extension of vase life in chrysanthemum by AKG is the result of an integrated, multi-level regulatory mechanism. AKG improves water relations, enhances antioxidant defenses, preserves membrane integrity, stimulates secondary metabolite accumulation, and suppresses ethylene biosynthesis at both transcriptional and enzymatic levels. These coordinated effects collectively delay the physiological and molecular events leading to petal senescence. The findings of this study highlight AKG as a promising, metabolically based preservative for extending the postharvest longevity and commercial value of ethylene-sensitive ornamental crops.

## Conclusion

In conclusion, this study demonstrates that exogenous AKG treatment effectively extends the postharvest vase life and maintains the ornamental quality of cut chrysanthemum flowers. The application of 5 mM AKG mitigates senescence by optimizing the postharvest water balance, reducing reactive oxygen species (ROS) accumulation via an enhanced enzymatic and non-enzymatic antioxidant defense system, and significantly suppressing the transcriptional expression of key ethylene biosynthesis genes (*CmACS1* and *CmACO1*). These findings highlight the scientific and economic potential of AKG as a safe, cost-effective, and eco-friendly metabolic intermediate for postharvest preservation in the floriculture industry. Future research should focus on exploring the broader molecular signaling networks and proteomic shifts induced by AfKG during flower senescence. Additionally, evaluating its synergistic effects when combined with other green preservation technologies under large-scale commercial transit conditions remains a valuable avenue for prospective investigations.

## Data Availability

The data that support the findings of this study are available from the corresponding author upon reasonable request. There are no restrictions on data availability.
